# Screening of drug targets for tuberculosis on the basis of transcription factor regulatory network and mRNA sequencing technology

**DOI:** 10.3389/fmolb.2024.1410445

**Published:** 2024-05-22

**Authors:** Shuai Wang, Na Yan, Yue Yang, Li Sun, Yingxin Huang, Jian Zhang, Guangyu Xu

**Affiliations:** ^1^ Department of Infectious Disease, Changchun Infectious Disease Hospital, Changchun, China; ^2^ College of Pharmacy, Beihua University, Jilin, Jilin, China

**Keywords:** tuberculosis, drug target, mRNA sequencing, transcription factor, regulatory network

## Abstract

**Background:**

Tuberculosis is a worldwide epidemic disease, posing a serious threat to human health. To find effective drug action targets for *Mycobacterium tuberculosis*, differentially expressed genes in tuberculosis patients and healthy people were screened by mRNA sequencing in this study. A total of 556 differentially expressed genes in tuberculosis patients and healthy people were screened out by mRNA sequencing technology. 26 transcription factors and 66 corresponding target genes were screened out in the AnimalTFDB 3.0 database, and a transcription factor regulatory network was constructed.

**Results:**

Three key transcription factors (TP53, KLF5 and GATA2) and one key gene (AKT1) were screened as new potential drug targets and diagnostic targets for tuberculosis by MCODE cluster analysis, and the key genes and key transcription factors were verified by RT-PCR. Finally, we constructed the and a key factor and KEGG signaling pathway regulatory network to clarify the possible molecular pathogenesis of tuberculosis.

**Conclusion:**

This study suggested *M. tuberculosis* may activate the AKT1 gene expression by regulating transcription factors TP53, KLF5, and GATA2, thus activating the B cell receptor signaling pathway to induce the infection and invasion of *M. tuberculosis*. AKT1, TP53, KLF5, and GATA2 can be used as new potential drug targets for tuberculosis.

## 1 Introduction

Tuberculosis is one of the most deadly infectious diseases in the world, and about one-quarter of the world’s population has been infected with *Mycobacterium tuberculosis* (Wilkins et al., 2022). According to the latest World Health Organization (WHO) report, there were approximately 10.6 million new cases and 1.3 million deaths worldwide in 2022 (Mi et al., 2024). The incidence and mortality of tuberculosis are still high worldwide, although its prevention and treatment have cost a lot (Drain et al., 2018; Huang et al., 2019). The main reason is the long-term incubation of tuberculosis and the delay in the emergence of new and effective anti-tuberculosis drugs (Venketaraman et al., 2015; Cole, 2016). Therefore, the research on new drug targets for tuberculosis is the focus of developing anti-tuberculosis drugs (Gashaw et al., 2011).

Drug targets refer to the binding sites of drugs *in vivo*, including biological macromolecules such as gene sites, receptors, enzymes, ion channels and nucleic acids. The key to modern new drug research and development is first to find, determine and prepare drug screening targets (Eder and Herrling, 2016). Transcription factors are important molecules that control gene expression and the convergence point of multiple signal pathways in eukaryotic cells, and play an important role in the function of cells and the healthy development of the body (Vaquerizas et al., 2009; Papavassiliou and Papavassiliou, 2016). Transcription factors and their corresponding target genes construct a corresponding regulatory network, and the key factors in this regulatory network are the targets of drug action or clinical diagnosis. Therefore, new potential drug targets for tuberculosis can be explored by constructing a transcription factor regulatory network for tuberculosis.

In this study, differentially expressed genes were screened in tuberculosis patients and healthy people by using mRNA sequencing technology. The transcription factors of the differentially expressed genes were screened in the AnimalTFDB 3.0 database (Hu W. et al., 2019) to construct a regulatory network, then the key targets and their related pathways were further screened by MCODE cluster analysis and Kyoto Encyclopedia of Genes and Genomes (KEGG) pathway analysis (Kanehisa et al., 2017) and the pathogenesis of tuberculosis and its potential new drug targets were analyzed.

## 2 Materials and methods

### 2.1 Data source and grouping

This study was approved by the Ethics Committee of Jilin Provincial Tuberculosis Hospital. In this study, 10 patients with pulmonary tuberculosis diagnosed in clinics were included (case group), and 10 healthy volunteers were taken as the normal control group (control group). After signing the written informed consent approved by the Ethics Committee of Jilin Provincial Tuberculosis Hospital, peripheral blood samples from the patients and volunteers were collected, and there were 10 sequencing samples in each group.

The sequences generated in the present study are available through the SRA Sequence Read Archive (https://www.ncbi.nlm.nih.gov/bioproject/PRJNA876021).

### 2.2 mRNA sequencing

The quality of total RNA extracted and purified from the whole blood samples was controlled by using Agilent Bioanalyzer 2100 (Agilent Technologies, United States). The RNA with RIN ≥ 7.0 was used in the study to ensure the construction of a high-quality downstream Total RNA-Seq library. The double-stranded cDNA was purified by using Agencourt AMpure XP magnetic beads. The concentration of cDNA in the library was quantified with an Invitrogen Qubit 3.0 Spectrophotometer (Thermo Fisher Scientific, United States) and the size distribution of library fragments was determined with an Agilent 2100 Bioanalyzer, and finally, the library was sequenced by 2 × 150 bp double-ended sequencing.

### 2.3 Construction of PPI network

The differentially expressed genes were input into the STRING (https://string-db.org/) database (Szklarczyk et al., 2021), and the research species was selected as Homes sapiens and the free nodes were removed to construct a protein-protein interactions (PPI) network of the differentially expressed genes. The CytoNCA plug-in (Zhang et al., 2012) for the analysis of network centrality analysis in Cytoscape 3.9.1 was used to sort the PPI networks according to Degree.

### 2.4 Screening of transcription factors

The differentially expressed genes were input into the AnimalTFDB 3.0 database (https://guolab.wchscu.cn/AnimalTFDB4//#/) (Tang et al., 2015), and the research species was selected as *Homo sapiens*, and the transcription factors with specific binding sites were selected as the research objects.

### 2.5 Construction of transcription factor regulatory network

Twenty transcription factors (TF) and 66 target genes were predicted to combine the total of 165 TF-to-target pairs (Supplementary Table S1). The relation obtained from the analysis of the differential co-expression was mapped to the human transcription factors and target gene pairs to obtain transcription regulation pairs. Finally, Cytoscape3.9.1 software was used for plotting.

### 2.6 MCODE cluster analysis

MCODE, a density-based algorithm for multi-component protein complexes, can grasp the correlation between targets as a whole, and is considered to be a module division method with a lower entropy value compared with the direct screening of key targets by using the average value and medium centrality (Bader and Hogue, 2003). The transcription factor regulatory network was imported into Cytoscape 3.9.1 software, and the MCODE plug-in was used to further screen the possible drug targets of tuberculosis and its diagnostic targets.

### 2.7 GO function annotation enrichment analysis and KEGG pathway enrichment analysis

DAVID (Database for Annotation Visualization and Integrated Discovery) database (Dennis et al., 2003) was used for the Gene Ontology (GO) function annotation enrichment analysis and Kyoto Encyclopedia of Genes and Genomes (KEGG) pathway analysis (Kanehisa et al., 2017) on the screened differential genes. According to the GO significance reflected by the differentially expressed genes (*p* < 0.05), the differentially expressed genes were further analyzed from the functional perspective.

### 2.8 RT-qPCR verification

Total RNA of peripheral blood samples was extracted by the TRIzol (Life Technologies) method according to manufacturer’s instructions. The RNA will then be transcribed into cDNA. The RT-qPCR program is set as follows: Keep at 95°C for 30 s; Denatured 95°C 15 s and anneal or extend at 60°C 1 min in 40 cycles. Primers are shown in Table 1.

**TABLE 1:** Primers for RT-qPCR.

Primers	Forward primer sequence	Reverse primer sequence
AKT1	AGG​AGA​TGG​ACT​TCC​GGT​CG	CAA​ACT​CGT​TCA​TGG​TCA​CGC
TP53	GCG​CTT​CGA​GAT​GTT​CCG​AG	ATG​GCG​GGA​GGT​AGA​CTG​AC
KLF5	ACT​GCG​ATT​ACC​CTG​GTT​GC	TCC​CAG​GTA​CAC​TTG​TAT​GGC​T
GATA2	TAT​GGC​GCC​GAA​ACG​CCA​A	GGT​CAG​TGG​CCT​GTT​AAC​ATT​GTG

## 3 Results

### 3.1 Screened differentially expressed genes

**FIGURE 1:**
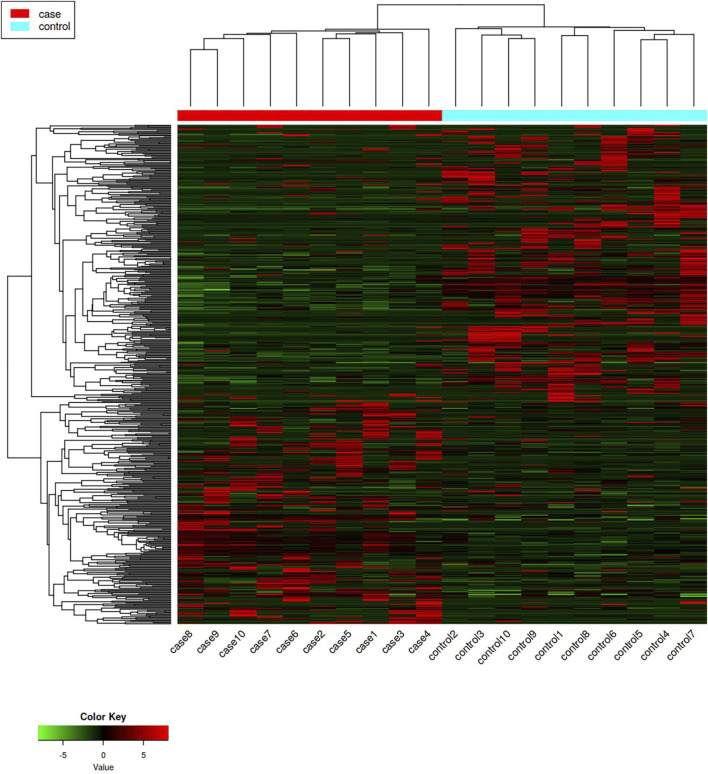
Hotspot map of mRNA sequencing results. The red represents upregulated differentially expressed genes, and the green represents downregulated differentially expressed genes.

**FIGURE 2:**
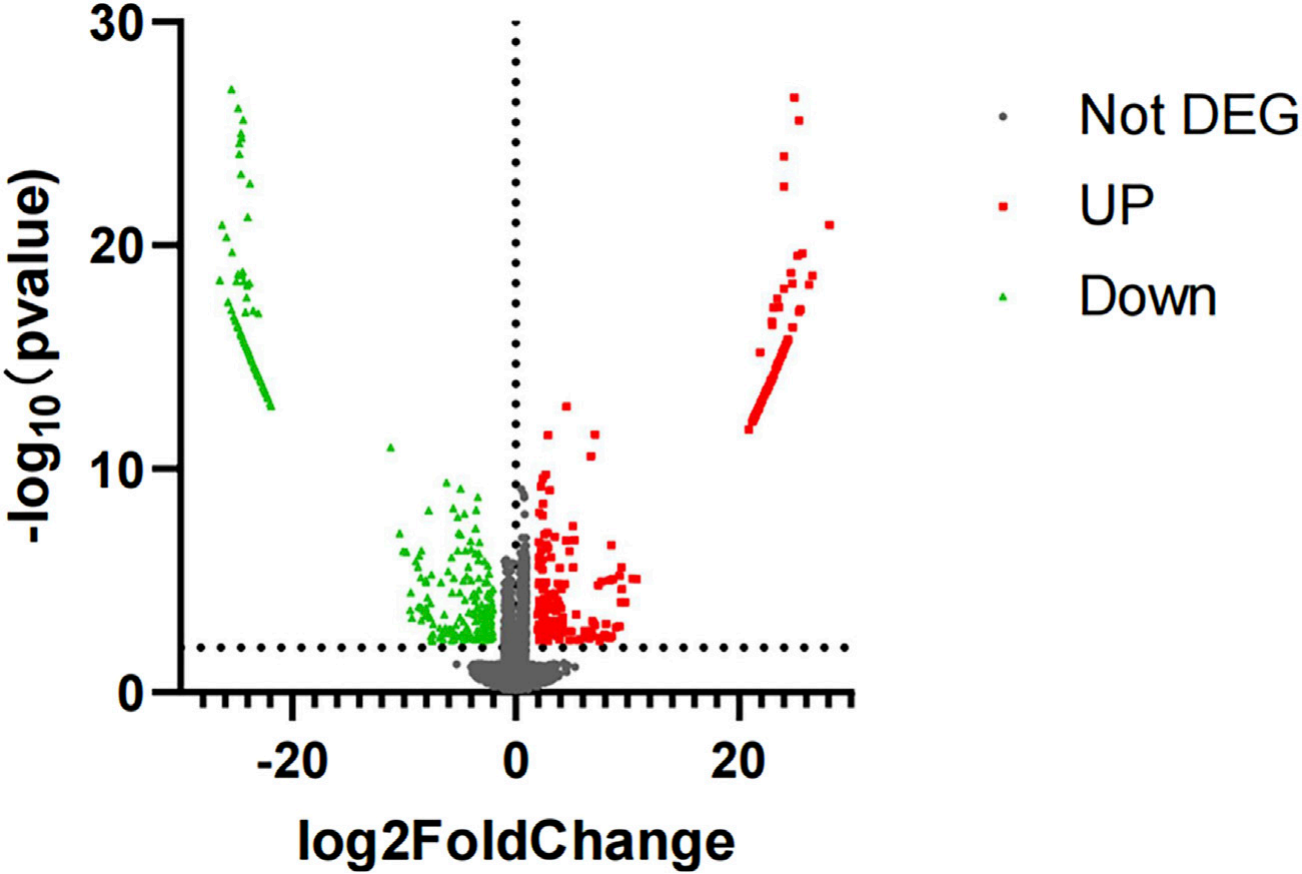
Volcano plot of mRNA sequencing. The red represents upregulated differentially expressed genes, and the green represents downregulated differentially expressed genes.

Differentially expressed genes in the case group and control group were screened with *p*-value < 0.005 and |log2 (fold change)| ≥ 2 as conditions, in which 556 differentially expressed genes were screened out (Supplementary Table S2), including 256 upregulated differentially expressed genes and 300 downregulated differentially expressed genes (Figures 1, 2).

### 3.2 Construction of PPI regulatory network of differentially expressed genes

**FIGURE 3:**
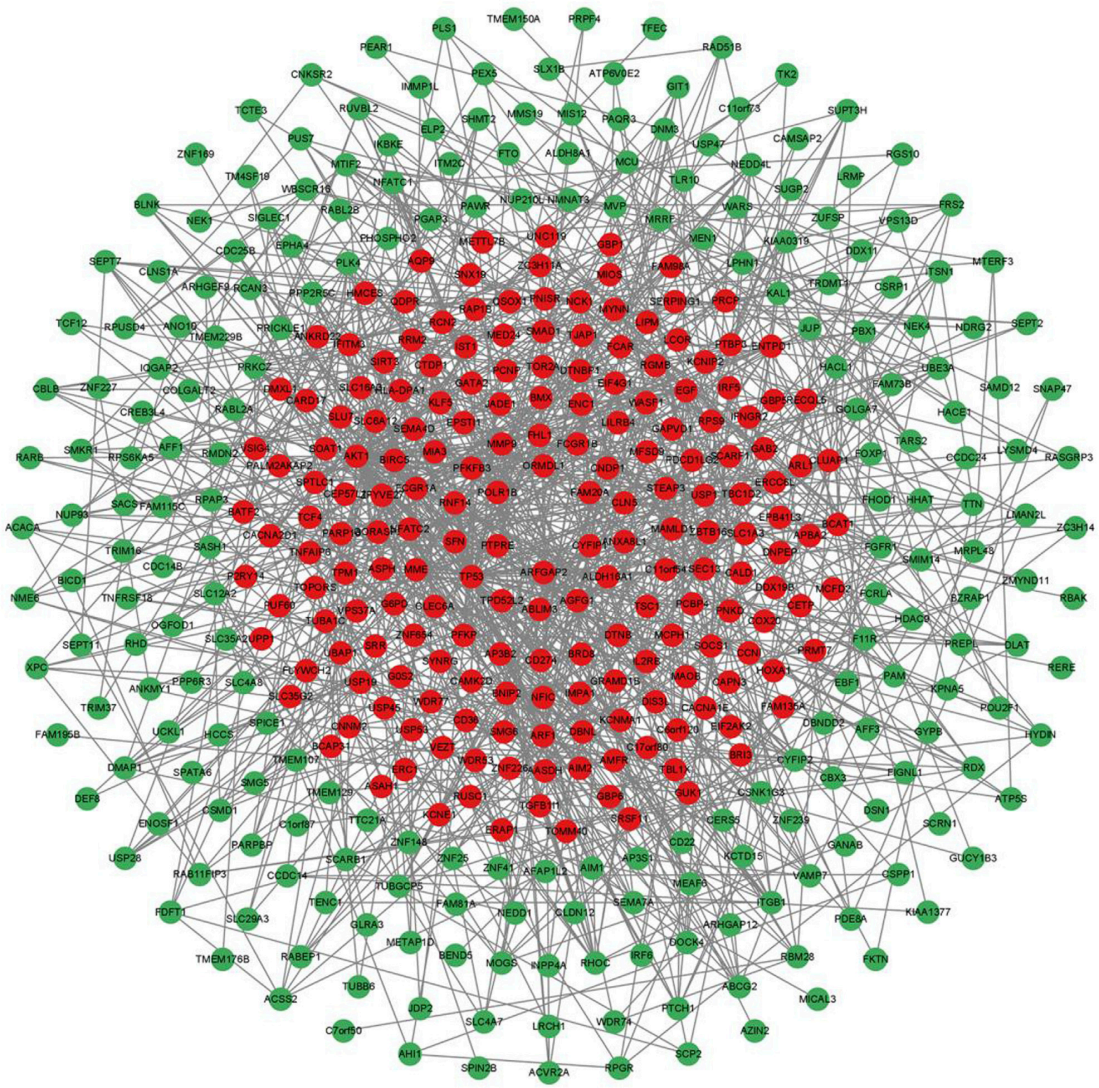
PPI regulation network of 430 important differentially expressed genes. The green nodes are the downregulated expressed genes, and the red nodes are the upregulated expressed genes.

We imported 556 differentially expressed genes into the STING database, then the free nodes deviating from the main network were removed, and finally the PPI regulatory network of 430 important genes was obtained (Figure 3). They were ranked according to Degree (Yang et al., 2019), and the top 10 important genes were AKT1, TP53, EGF, NCK1, ARF1, CD274, ITGB1, PRKCZ, RHOC, and PLK4 (Table 2).

**TABLE 2:** Differentially expressed genes in the top 10 order of Degree in the PPI regulatory network.

No..	Gene	Gene ID	Degree	Description
1	AKT1	207	138	AKT serine/threonine kinase 1
2	TP53	7157	118	tumor protein p53
3	EGF	1950	44	epidermal growth factor
4	NCK1	4690	42	NCK adaptor protein 1
5	ARF1	375	38	ADP ribosylation factor 1
6	CD274	29126	32	CD274 molecule
6	ITGB1	3688	32	integrin subunit beta 1
8	PRKCZ	5590	30	protein kinase C zeta
8	RHOC	389	30	ras homolog family member C
10	PLK4	10733	26	polo like kinase 4

### 3.3 Transcription factor regulatory network diagram

**FIGURE 4:**
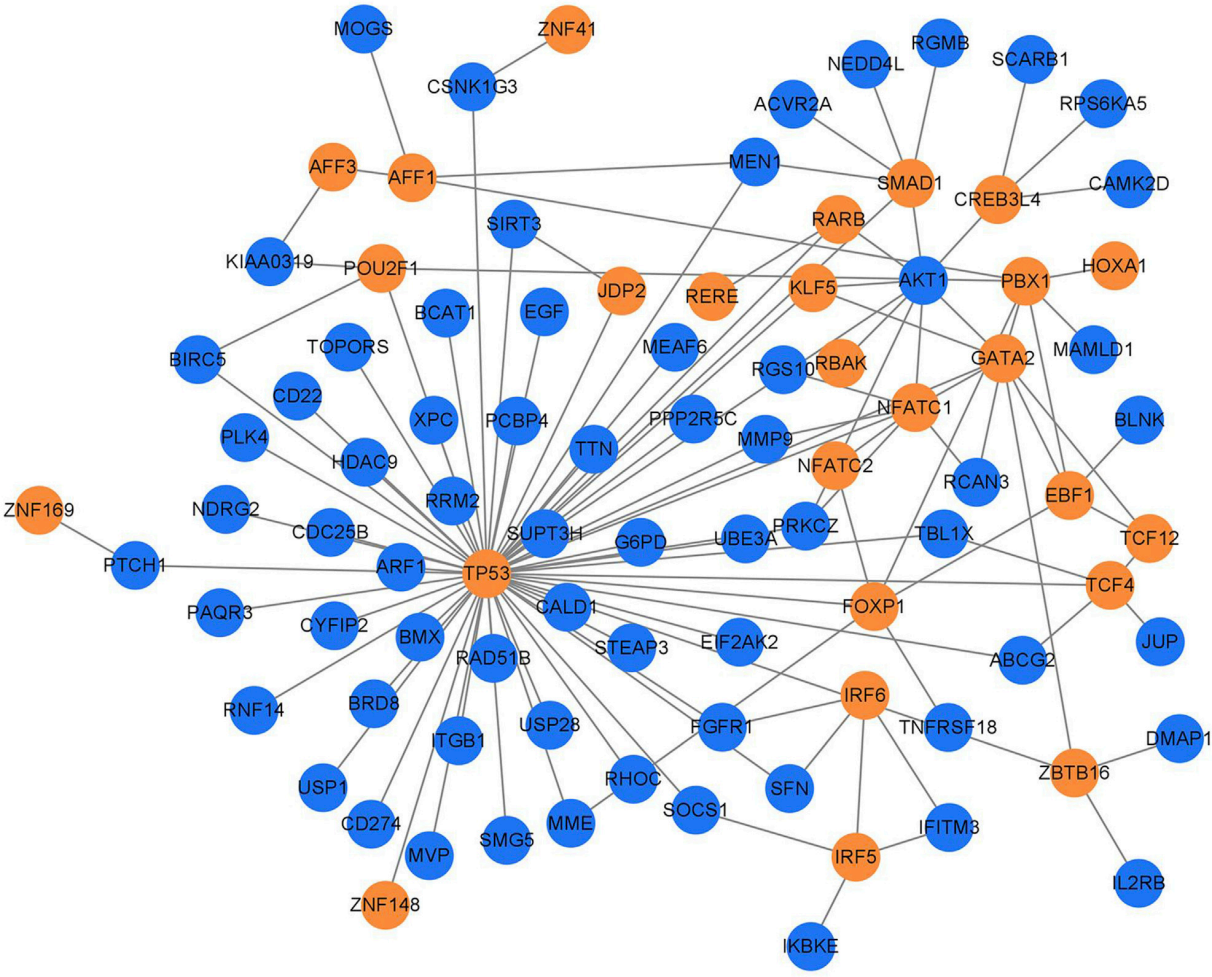
TB transcription factor regulatory network. The yellow circles represent the transcription factors, and the blue circles represent the regulated target genes.

Transcription factors of 430 important genes in the PPI regulatory network were screened in the AnimalTFDB 3.0 database, and 26 transcription factors and 66 target genes regulated by them were screened out (Figure 4; Table 3).

**TABLE 3:** 26 Transcription factors.

TF	Target gene number	Gene ID	Description
TP53	59	7157	tumor protein p53
GATA2	9	2624	GATA binding protein 2
NFATC1	8	4772	nuclear factor of activated T cells 1
PBX1	7	5087	PBX homeobox 1
SMAD1	6	4086	SMAD family member 1
FOXP1	6	27086	forkhead box P1
EBF1	5	1879	EBF transcription factor 1
TCF4	5	6925	transcription factor 4
AFF1	4	4299	ALF transcription elongation factor 1
CREB3L4	4	148327	cAMP responsive element binding protein 3 like 4
IRF5	4	3663	interferon regulatory factor 5
IRF6	4	3664	interferon regulatory factor 6
NFATC2	4	4773	nuclear factor of activated T cells 2
POU2F1	4	5451	POU class 2 homeobox 1
ZBTB16	4	7704	zinc finger and BTB domain containing 16
KLF5	3	688	Kruppel like factor 5
RARB	3	5915	retinoic acid receptor beta
TCF12	3	6938	transcription factor 12
AFF3	2	3899	ALF transcription elongation factor 3
JDP2	2	122953	Jun dimerization protein 2
HOXA1	1	3198	homeobox A1
RBAK	1	57786	RB associated KRAB zinc finger
RERE	1	473	arginine-glutamic acid dipeptide repeats
ZNF148	1	7707	zinc finger protein 148
ZNF169	1	169841	zinc finger protein 169
ZNF41	1	7592	zinc finger protein 41

It was found in the transcription factor regulation network (Figure 4) that 10 target genes were regulated by more than 2 transcription factors (Table 4), among which TP53, AKT1, GATA2, and PBX1 were the targets regulated by the most transcription factors, 11, 9, 7, and 6 transcription factors, respectively.

**TABLE 4:** Genes regulated by transcription factors.

Genes regulated by TF	No. of TF regulating target genes	Node
TP53	11	59
AKT1	9	9
GATA2	7	9
PBX1	6	7
FOXP1	4	6
EBF1	4	5
TCF12	3	3
PRKCZ	3	3
NFATC1	3	8
MEN1	3	3

TF, transcription factor.

### 3.4 Key factors screened by MCODE cluster analysis

**FIGURE 5:**
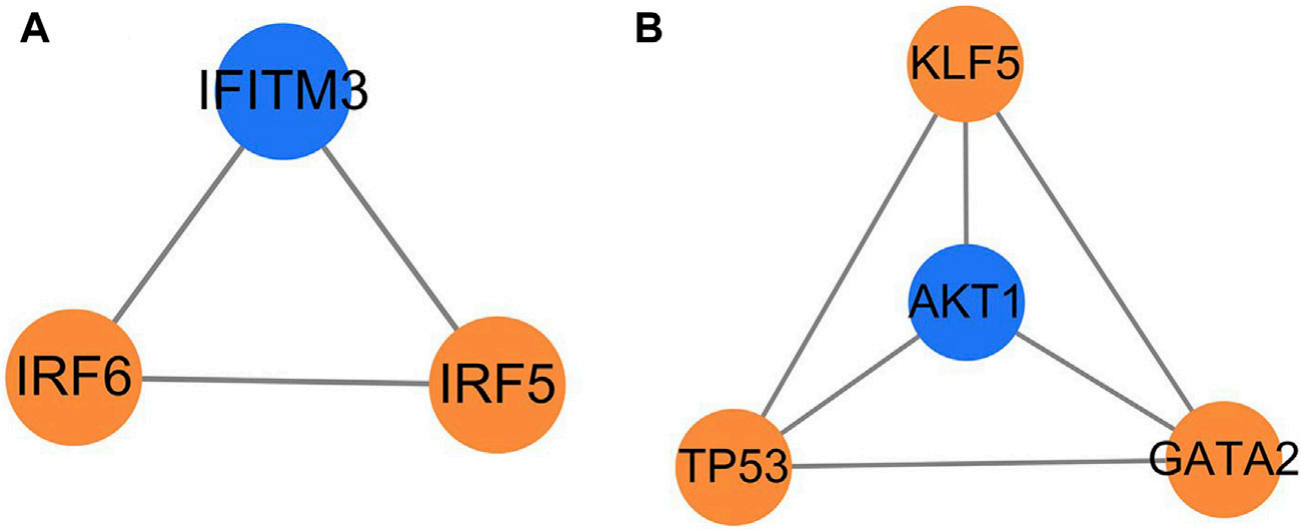
Core regulation sub-network. The yellow represents the transcription factors, and the blue represents the target genes; **(A)** score = 3 and **(B)** score = 4.

The MCODE cluster analysis on 26 transcription factors and 66 target genes regulated by them in the transcription factor regulatory network was also performed with the plug-in in Cytoscape 3.9.1 software, and two core regulatory sub-networks were obtained (Figures 5A, B). The core regulatory sub-network with the highest score (score = 4) was selected for research (Figure 5B), and four key factors (AKT1, TP53, KLF5, and GATA2) were obtained, of which TP53, GATA2, and KLF5 were the key transcription factors, and AKT1 was the key target gene regulated by transcription factors.

### 3.5 GO cluster analysis

**FIGURE 6:**
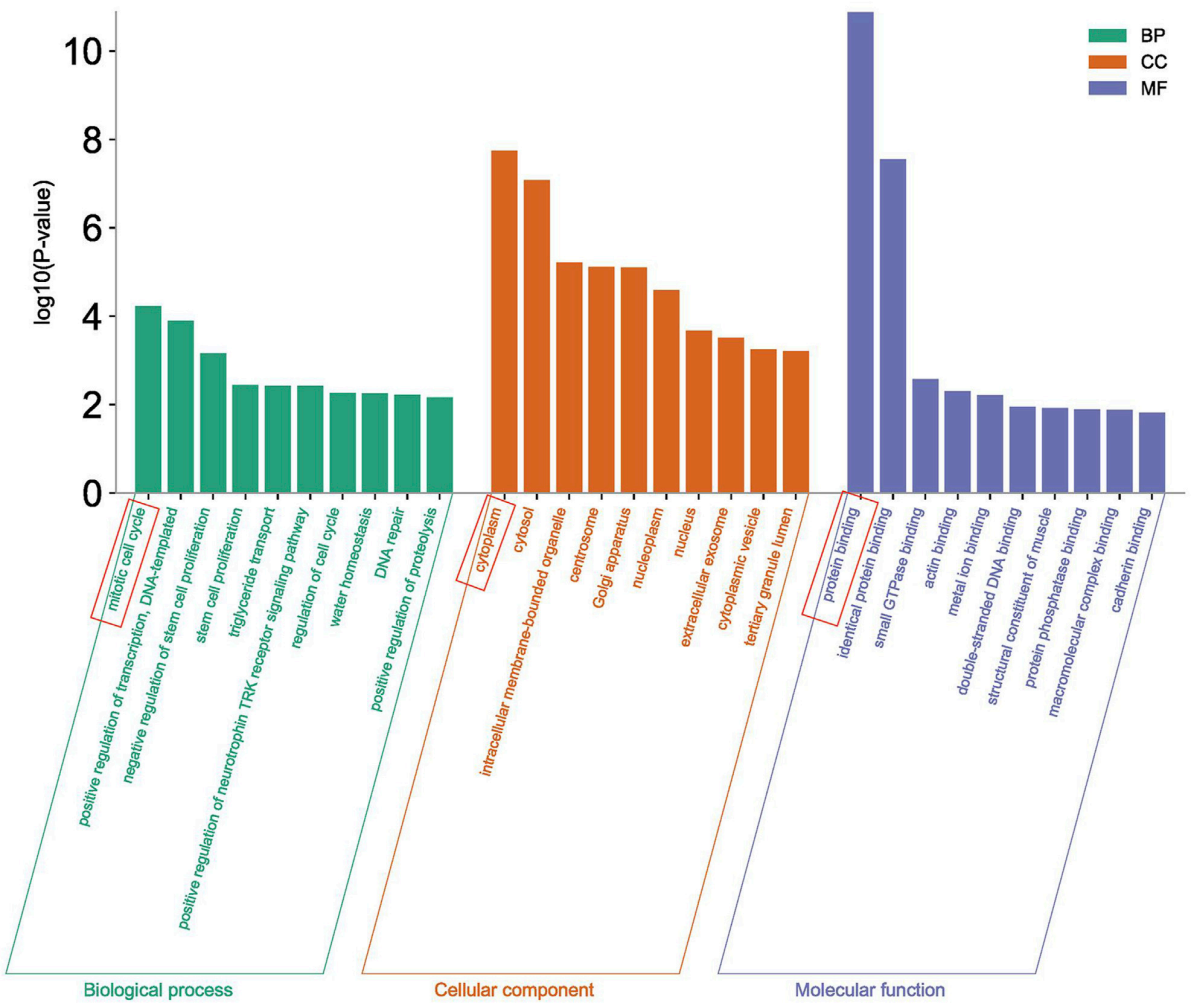
GO enrichment analysis.

The Gene Ontology analysis of 430 differentially expressed genes was performed in the DAVID database, and a total of 222 items were obtained. Among them, 121 items were related to Biological Process (BP), of which the item with the highest *p*-value significance was the mitotic cell cycle, 61 items were related to the Cellular Component (CC), of which the item with the highest *p*-value significance was cytoplasm, and 40 items were related to Molecular Function (MF), of which the item with the highest *p*-value significance was protein binding (Figure 6).

### 3.6 KEGG channel analysis

**FIGURE 7:**
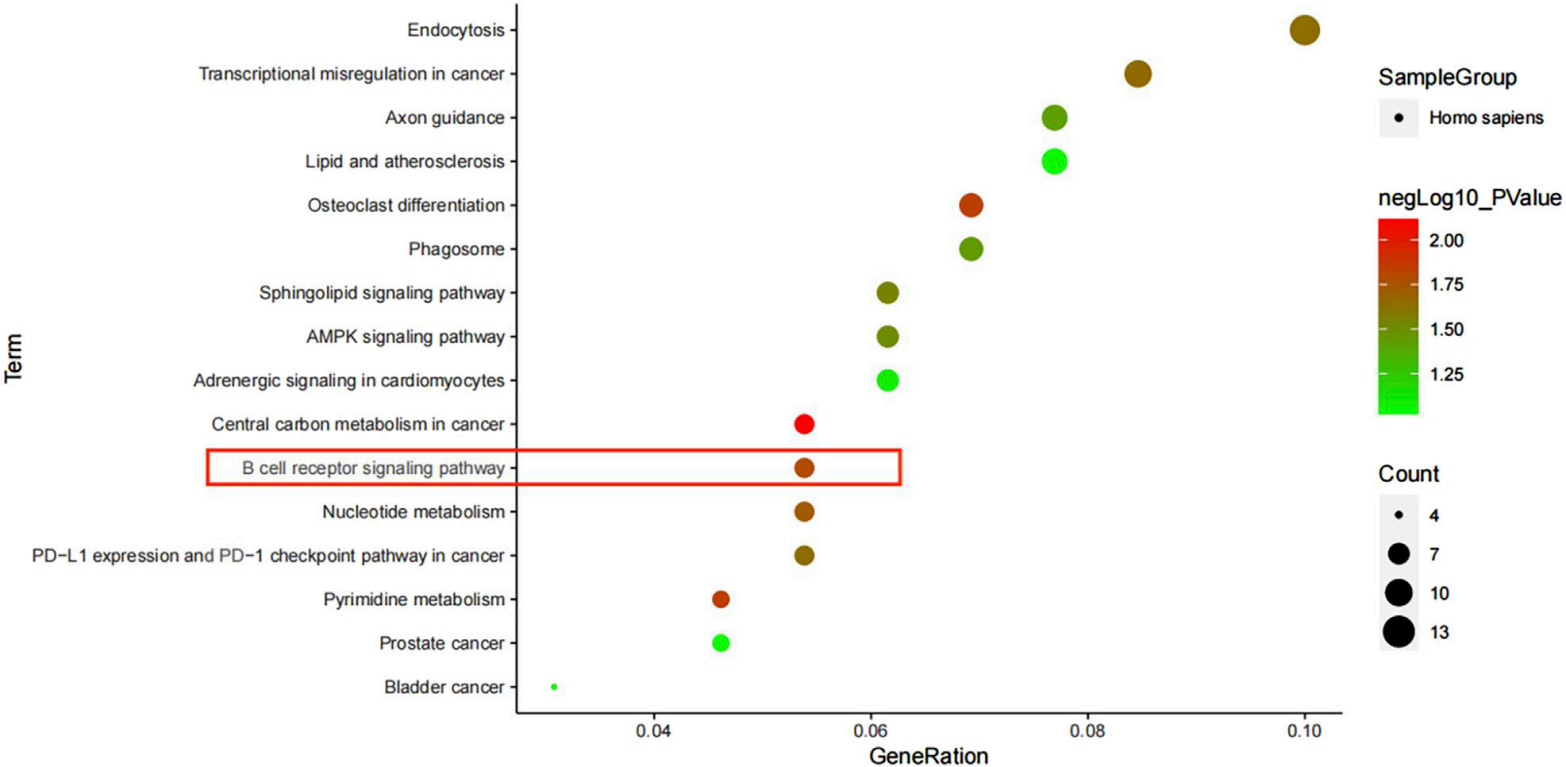
KEGG pathway enrichment analysis.

We analyzed 430 differentially expressed genes in the KEGG database and obtained 16 signal pathways (*p* < 0.05), mainly related to immunity and cancer. Among them, the most significant pathway was the central carbon metabolism in cancer, and there were three signal pathways (B cell receiver signaling pathway, Sphingolipid signaling pathway and AMPK signaling pathway), of which B cell receiver signaling pathway was the most significant signal pathway, mainly related to immunity (Figure 7).

### 3.7 Construction of functional relationship network diagram of key genes

**FIGURE 8:**
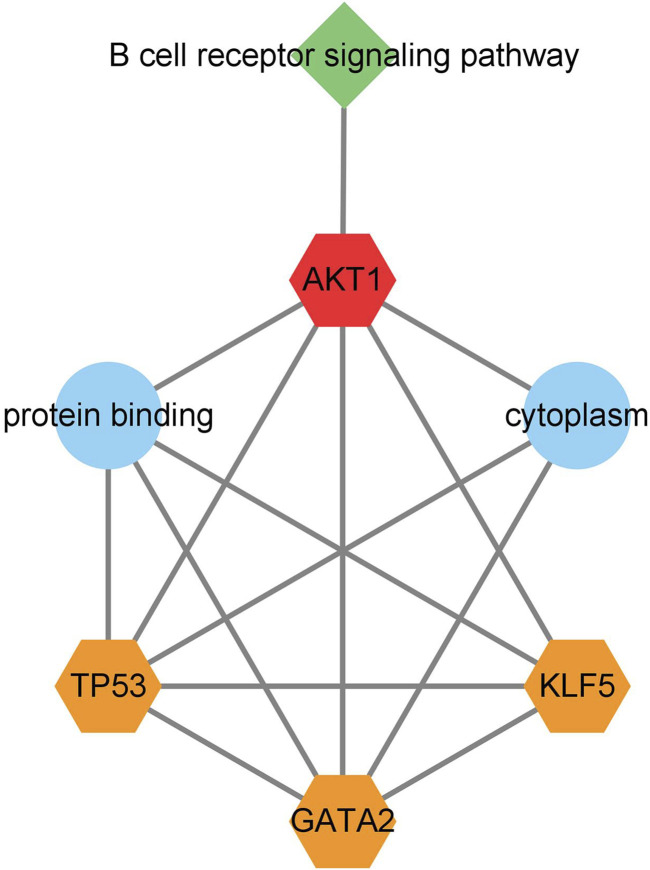
Network diagram of the relationship between network nodes. The orange pentagons represent transcription factors, the red pentagons represent target genes regulated by transcription factors, the blue circles represent Gene Ontology, and the green quadrangles represent the B cell receptor signaling path.

A network diagram of the relationship between network nodes (Figure 8) was constructed by combining the three key transcription factors (TP53, GATA2, and KLF5), one key gene (AKT1) and the related pathway (B cell receiver signaling pathway) previously screened. It was found in the network diagram of the relationship between network nodes that AKT1, TP53, KLF5, and GATA2 were all related to the protein binding function, and AKT1, TP53 and GATA2 were also related to the cytoplasm function. The target gene AKT1 mainly played a regulatory role in the immune-related pathway B cell receiver signaling pathway, so it was inferred that *M. tuberculosis* may activate the expression of the AKT1 gene by regulating the transcription factors TP53, KLF5 and GATA2, thus leading to the infection and invasion of *M. tuberculosis* through B cell receiver signaling pathway.

### 3.8 RT-qPCR verification experiment

**FIGURE 9:**
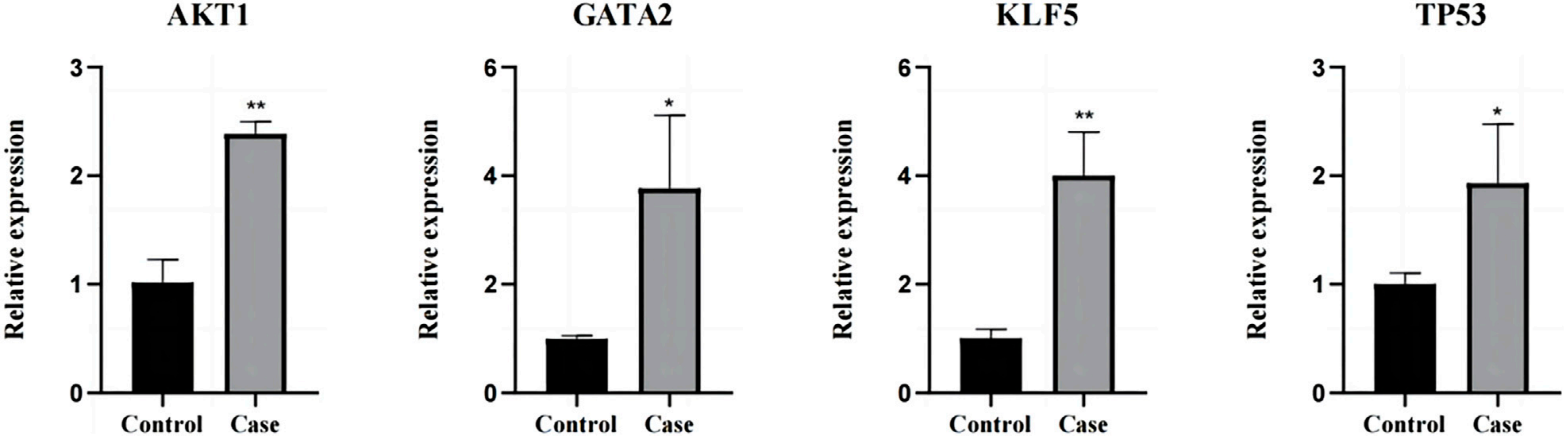
The results were verified by RT-qPCR. *: *p* < 0.05, **: *p* < 0.01.

The four key factors (AKT1, TP53, KLF5, and GATA2) screened were verified by RT-PCR. AKT1, TP53, KLF5, and GATA2 were all upregulated genes, and the results of RT-PCR experiment were consistent with the results of mRNA sequencing (Figure 9).

## 4 Discussion

In recent years, tuberculosis has made a comeback, with a significant rise in its incidence and mortality, and the resistance of *M. tuberculosis* to anti-tuberculosis drugs commonly used at present has become increasingly serious, which has become a thorny problem in the clinical treatment of tuberculosis (Suárez et al., 2019; Olivença et al., 2022). In the past 30 years, no new and efficient anti-tuberculosis drugs have been developed. Therefore, it is urgent to find potential targets of new anti-tuberculosis drugs for studying and developing new anti-tuberculosis drugs to achieve effective control of tuberculosis (Rode et al., 2019). In this study, 556 differentially expressed genes were screened out by mRNA sequencing, and 430 important differentially expressed genes were identified by constructing a PPI regulatory network and removing free genes. Twenty-six transcription factors and 66 target genes regulated by them were identified from 430 important differentially expressed genes through the AnimalTFDB 3.0 database.

A transcription factor regulatory network of 26 transcription factors and 66 target genes regulated by them was constructed. It was found by the analysis of the transcription factor regulatory network that the target genes corresponding to TP53 were the most common (59), and its Degree value ranked second in the PPI regulatory network. TP53 is the coding gene of the tumor suppressor gene p53 protein, located on human chromosome 17 (Hu H. et al., 2019). TP53 wild-type mutation will lead to apoptosis of cancer cells, while TP53 mutant will increase the risk of cancer, so that the mutation status of TP53 may be a biomarker to predict the response of different cancer types to cancer immunotherapy (Li et al., 2020). In addition, some studies have pointed out that TP53 can regulate human immune function and affect the host immune system (Yuan et al., 2018). GATA2 is the second most common transcription factor (9), corresponding to the target gene. GATA2 is a zinc-finger transcription factor expressed in human hematopoietic stem cells and various hematopoietic progenitor cells (Oleaga-Quintas et al., 2021). There are also many target genes corresponding to the transcription factor GATA2 (9), and the GATA2 gene is believed to regulate the ontogeny and function of monocytes, macrophages, dendritic cells, B cells and NK cells (Yuag et al., 2017; Monif et al., 2018). Therefore, we find that both TP53 and GATA2 are closely related to the body’s immune system.

The analysis of target genes regulated by transcription factors showed that TP53, AKT1, GATA2 and PBX1 were the four target genes most regulated by transcription factors, regulating 11, 9, 7 and 6 target genes, respectively. Our previous study has shown that genes TP53 and GATA2 are very important transcription factors and are closely related to immune function. AKT1 is a serine/threonine protein kinase, also known as Akt kinase. Akt can regulate the development and function of innate immune cells such as neutrophils, macrophages and dendritic cells, which play an important role in the regulation of immune cells (Xia et al., 2020). AKT1 plays a key role in the process of controlling the growth of *M. tuberculosis* cells and is a key kinase involved in controlling the growth of *M. tuberculosis* cells. AKT1 inhibitors have the potential to be used as antibiotics to treat tuberculosis (Wang et al., 2010). The PBX1 gene promotes the early development of NK cells by directly up-regulating the expression of Nfil3 (Xu et al., 2020). PBX1 is required to maintain the self-renewal of hematopoietic stem cells during the development of the immune system. PBX1 deficient embryonic stem cells cannot produce lymphoid progenitor cells, leading to the loss of B and NK cells as well as the impaired development of T cells (Niu et al., 2017; Gu et al., 2023). Therefore, these four target genes that are most regulated by transcription factors are also closely related to the immune function of the body.

Through MCODE cluster analysis, a core sub-network of the transcription factor regulatory network was constructed, and four important nodes, AKT1, TP53, KLF5, and GATA2 were screened out. As described above, AKT1 is an important gene regulated by transcription factors, TP53 and GATA2 are important transcription factors, and AKT1, TP53, and GATA2 are all related to the immune function of the body. The MCODE cluster analysis showed that transcription factor KLF5 was closely related to other key genes (Figure 5B) although it only regulated three target genes (Table 3). Some studies have shown that KLF5 plays an important role in the differential regulation of angiogenesis progress induced by *M. tuberculosis*, closely related to the pathogenesis of pulmonary tuberculosis, and the expression of the KLF5 gene has also been verified by immunofluorescence imaging in these studies (Mukherjee et al., 2022). It can be inferred that tuberculosis may activate the expression of the AKT1 gene by regulating the transcription factors TP53, KLF5, and GATA2, thus leading to the infection and invasion of *M. tuberculosis*.

The GO analysis and KEGG analysis of 430 important differentially expressed genes screened were also performed in this study. The GO analysis showed that the enrichment of the mitotic cell cycle was the most significant in the biological processes (BP), and it is well known that mitosis is an important process to maintain the normal growth and development of individuals. It has been indicated in some studies that the mitotic index of tuberculosis patients who have received treatment with anti-tuberculosis drugs will improve (Jaju et al., 1983). In the cell components (CC), the enrichment of cytoplasm was the most significant. The cytoplasm is the main place for metabolism and plays a regulatory role in the nucleus. In the molecular functions (MF), the enrichment of protein binding was the most significant. The GO enrichment analysis results suggest that *M. tuberculosis* may play a regulatory role in the nucleus by changing the function of the main metabolic sites, thus affecting the protein binding function.

In the KEGG pathway analysis of differential genes, we screened out 16 pathways, of which central carbon metabolism in cancer was the most significant. Tuberculosis and cancer are two diseases that tend to produce resistance to the host immune system, with certain similarities in the regulation of immune response (Bickett and Karam, 2020). B cell receiver signaling pathway is one of the most significant signal pathways, mainly related to immunity (Sun et al., 2020; Tanaka and Baba, 2020). The key gene AKT1 can regulate this pathway, and the gene AKT1 is one of the four key nodes we screened out. Therefore, the gene AKT1 is likely to be the key gene to activate the B cell receiver signaling pathway.

## 5 Conclusion

Four key genes and a pathway closely related to tuberculosis were screened out by constructing the tuberculosis transcription factor network, of which TP53, KLF5, and GATA2 are transcription factors and AKT1 is the target gene. *Mycobacterium tuberculosi*s may activate the expression of the AKT1 gene by regulating the transcription factors TP53, KLF5 and GATA2, thus leading to the infection and invasion of *M. tuberculosis* through the B cell receptor signaling pathway.

## Data Availability

The data presented in this study are deposited in the NIH SRA repository, accession number: PRJNA876021.
